# Liver Tissue Mapping in Transfusion-Dependent β-Thalassemia: Reproducibility and Clinical Insights from Multiparametric MRI

**DOI:** 10.3390/diagnostics15233085

**Published:** 2025-12-04

**Authors:** Antonella Meloni, Riccardo Bisi, Vincenzo Positano, Aldo Carnevale, Nicola Pegoraro, Laura Pistoia, Anna Spasiano, Elisabetta Corigliano, Antonella Cossu, Emanuela De Marco, Ilaria Fotzi, Petra Keilberg, Alberto Clemente, Alberto Cossu

**Affiliations:** 1 Bioengineering Unit, Fondazione G. Monasterio CNR-Regione Toscana, 56124 Pisa, Italy; positano@ftgm.it; 2 Department of Translational Medicine, University of Ferrara, 44121 Ferrara, Italy; riccardo01.bisi@edu.unife.it (R.B.); aldo.carnevale@unife.it (A.C.); nicola01.pegoraro@edu.unife.it (N.P.); 3 Unità Operativa Complessa Ricerca Clinica, Fondazione G. Monasterio CNR-Regione Toscana, 56124 Pisa, Italy; laura.pistoia@ftgm.it; 4 Unità Operativa Semplice Dipartimentale Malattie Rare del Globulo Rosso, Azienda Ospedaliera di Rilievo Nazionale “A. Cardarelli”, 80131 Napoli, Italy; spasiano.anna@tiscali.it; 5 Ematologia Microcitemia, Ospedale San Giovanni di Dio—ASP Crotone, 88900 Crotone, Italy; elisabetta.corigliano@asp.crotone.it; 6 Ambulatorio Trasfusionale—Servizio Immunoematologia e Medicina Trasfusionale Dipartimento dei Servizi, Presidio Ospedaliero “San Francesco”, 08100 Nuoro, Italy; a.cossu.1@aslnuoro.it; 7 Unità Operativa Oncoematologia Pediatrica, Azienda Ospedaliero Universitaria Pisana—Stabilimento S. Chiara, 56126 Pisa, Italy; e.demarco@ao-pisa.toscana.it; 8 Struttura Organizzativa Complessa Oncologia, Ematologia e Trapianto di Cellule Staminali Emopoietiche, Meyer Children’s Hospital IRCCS, 50139 Firenze, Italy; ilaria.fotzi@meyer.it; 9 Department of Radiology, Fondazione G. Monasterio CNR-Regione Toscana, 56124 Pisa, Italy; petrakeilberg@monasterio.it (P.K.); clemente@ftgm.it (A.C.); 10 Unità Operativa Radiologia Universitaria, Azienda Ospedaliero-Universitaria “S. Anna”, 44124 Ferrara, Italy; csslrt@unife.it

**Keywords:** liver, relaxation times, mapping, iron overload

## Abstract

**Background/Objectives**: We measured hepatic T2*, T1, and T2 values in *N* = 81 transfusion-dependent thalassemia (TDT) patients to assess and compare their reproducibility, evaluate their correlations with demographics and clinical parameters, and explore their association with disease-related complications. **Methods:** All TDT patients (52 females, 38.13 ± 10.79 years), were enrolled in the Extension-Myocardial Iron Overload in Thalassaemia Network. The magnetic resonance imaging protocol (1.5 T) included: multi-echo gradient echo sequences for T2* relaxometry, modified look-locker inversion recovery (MOLLI) sequences for T1 mapping, and multi-echo fast-spin-echo (MEFSE) sequences for T2 mapping. **Results:** All three relaxation times demonstrated good intra- and inter-observer reproducibility and were significantly correlated with each other. Of the 59 patients with reduced T2*, 45 (76.3%) also had reduced T1, and 42 (71.2%) had reduced T2 values. Among 22 patients with normal T2*, 3 (13.6%) exhibited reduced T1. No patients showed increased T1, and only one had elevated T2. Liver relaxation times were not associated with gender or splenectomy status. All relaxation times inversely correlated with serum ferritin levels, while T2 and T2* inversely correlated with mean alanine aminotransferase levels. Cirrhosis and glucose metabolism alterations were associated with lower relaxation times. All three relaxation times effectively discriminated between the absence and presence of cirrhosis [areas under the curve (AUCs) with 95% confidence intervals (CIs): 0.85 (0.75–0.92) for T2*, 0.78 (0.68–0.87) for T1, and 0.92 (0.84–0.97) for T2]. T2* showed comparable accuracy to T1 and T2, while a significant difference was observed between T1 and T2 values. All liver relaxation times demonstrated similar diagnostic performance in identifying glucose metabolism alterations [AUCs with 95% CIs: 0.67 (0.55–0.77) for T2*, 0.69 (0.57–0.79) for T1, and 0.67 (0.56–0.77) for T2]. **Conclusions:** In TDT, a comprehensive assessment of hepatic relaxation times may enhance clinical monitoring and management of iron overload and its related complications.

## 1. Introduction

Transfusion-dependent β-thalassemia (TDT) is a severe inherited blood disorder resulting from mutations in the beta-globin gene, leading to reduced or absent synthesis of beta-globin chains. This imbalance disrupts normal hemoglobin production and causes ineffective erythropoiesis, resulting in severe, chronic anemia that necessitates lifelong regular blood transfusions [1,2]. While transfusions are essential for survival, they also introduce excess iron into the body—a burden exacerbated by the absence of a physiological mechanism for iron excretion [3,4]. Over time, this iron accumulates in vital organs, leading to progressive tissue damage and a wide spectrum of chronic, potentially life-threatening complications [5,6,7].

Cardiac involvement represents the primary cause of mortality [8,9] and cardiac magnetic resonance (CMR), due to its multiparametric potential [10,11], provides excellent characterization capabilities of the complex clinical picture affecting TDT patients [12]. More than 20 years ago, the T2* CMR technique emerged as a valid and reproducible method for the non-invasive detection of myocardial iron overload (MIO) [13]. This advancement has profoundly transformed patient management by allowing for more effective monitoring of disease progression and guiding therapy, ultimately improving survival outcomes [9,14,15]. The presence of iron deposits reduces not only T2* values but also T1 and T2 relaxation times, reflecting the paramagnetic effects of iron on tissue relaxation properties [16]. In TDT patients with borderline cardiac iron burden, native T1 mapping has demonstrated higher sensitivity than T2* imaging for the detection of MIO [17,18,19,20]. Nonetheless, T1 mapping is less specific for iron quantification, as concurrent inflammation, fat, and diffuse fibrosis may confound the early detection of iron accumulation [21]. T2 mapping, in contrast, was shown to provide additional value by enabling the assessment of myocardial edema indicative of inflammation [22].

The reduction in cardiac mortality in TDT has shifted the disease burden toward other organ complications [23] and hepatopathy is rising as a notable cause of mortality [24]. Hepatic iron overload is a critical factor in the pathogenesis of chronic liver disease, promoting the development of fibrosis and its progression to cirrhosis [25,26,27]. The prevalence of liver disease is also influenced by hepatitis B virus (HBV) and hepatitis C virus (HCV) chronic infections, which burdened transfusions before the advent of transfusion screening programs and to which TDT patients remain exposed in Countries where transfusion bio-safety standards are lower [28,29]. In addition to iron and infection-related toxicity, other liver complications are emerging. Metabolically dysfunction-associated steatotic liver disease (MASLD), formerly known as non-alcoholic fatty liver disease (NAFLD), is gaining prominence, largely due to the rising prevalence of obesity and diabetes mellitus (DM) in this population, conditions that are becoming more common due to the extended life expectancy of these patients [30]. These evolving risk factors highlight the need for comprehensive liver monitoring and early intervention strategies, including the use of non-invasive imaging techniques such as multiparametric MRI to assess liver iron, fat, and fibrosis.

Both T2* and T2 magnetic resonance imaging (MRI) techniques, when combined with appropriate calibration curves, have been shown to accurately assess liver iron concentration (LIC) and are used in clinical practice [31,32,33,34,35,36]. A small study involving 25 patients with hereditary hemochromatosis showed that T1 mapping was not more sensitive than the T2* technique in detecting biopsy-proven hepatic iron overload, while it provided additional insights into the potential presence of steatohepatitis or its coexistence with iron overload [37]. This study is in line with other studies always conducted in non-thalassemic populations, where T1 mapping has proven effective in detecting steatosis, fibrosis, and inflammatory activity [38,39,40,41]. In the same line, a study involving patients with hepatitis C-related chronic liver disease, but without iron accumulation or steatosis on random liver biopsy, demonstrated that varying grades of fibrosis exhibit statistically significant differences in T2 values, likely due to the increased inflammatory component that occurs prior to or alongside fibrosis [42]. To the best of our knowledge, studies assessing all three relaxation times in vivo in the human liver of TDT patients are currently lacking. Such studies may help clarify the potential benefits of a multiparametric strategy for iron overload assessment and tissue characterization. Additionally, examining the relationship of these relaxation times with complications such as cirrhosis or metabolic disturbances could provide valuable insight into their clinical significance and diagnostic utility, helping to identify the most effective imaging biomarkers.

This study measured hepatic T2*, T1, and T2 values in TDT patients to assess and compare their reproducibility, evaluate their correlations with demographics and clinical parameters, and explore their association with disease-related complications.

## 2. Materials and Methods

### 2.1. Study Population

The Extension-Myocardial Iron Overload in Thalassemia (E-MIOT) project is an Italian network constituted by 66 thalassemia centers and 15 validated MRI sites [43,44], connected through a centralized web-based database collecting all patients’ demographic, clinical, and instrumental data. According to the study protocol, all thalassemia patients perform a CMR T2* every 18 ± 3 months for MIO quantification.

We included in this retrospective cross-sectional study all consecutive patients with TDT who underwent CMR in the reference MR center of the E-MIOT Network (Pisa) between June 2018 and June 2022. During that interval, T1 and T2 mapping were routinely included in the CMR protocol. The left lobe of the liver was largely covered within the acquisition field of view and patients with a non-diagnostic T1 and/or T2 mapping acquisition pertaining to the liver were excluded. If a patient performed more than one CMR scan, only the first exam was included.

The study complied with the Declaration of Helsinki and was approved by the institutional ethics committee. All subjects and patients gave written informed consent to the protocol.

### 2.2. MRI Acquisition and Image Analysis

CMR exams were performed on a 1.5 T scanner (Signa Artist; GE Healthcare, Milwaukee, WI, USA) using a 30-element cardiac phased-array receiver surface coil with breath-holding and ECG-gating.

Three parallel short-axis slices (basal, medial, and apical) of the left ventricle (LV) were acquired in end-diastole by a multi-echo gradient echo (GRE) sequence [10 echo times (TEs) with echo spacing = 2.26 ms, flip angle (FA) = 25°, matrix = 192 × 192 pixels, slice thickness = 8 mm] for T2* relaxometry [45], a modified look-locker inversion recovery (MOLLI) sequence [scheme = 3(3s)3(3s)5, FA = 35°, TE = 1.6 ms, repetition time = 3.7 ms, matrix = 172 × 172 pixels, slice thickness = 8 mm] for T1 mapping [18], and a multi-echo fast-spin-echo (MEFSE) sequence [blood-prepulse, 4 TEs = 9.78, 34.22, 58.66, and 83.10 ms, echo train length = 20, matrix = 256 × 256 pixels, slice thickness = 8 mm] for T2 mapping [46].

Image analysis was performed by a single operator with 2 years of experience in abdominal MRI (R. B.) and the analysis accuracy was reviewed by a radiologist with >15 years of experience in abdominal MRI (A. C.).

T2* image analysis was performed using a custom-written and validated software (HIPPO MIOT^®^, Version 2.0, Consiglio Nazionale delle Ricerche and Fondazione Toscana Gabriele Monasterio, Pisa, Italy). In GRE images corresponding to the first or second TE, polygonal regions of interest (ROI) (size: 0.64 pixel counts/mm^2^) were manually drawn in the visible and detectable functionally independent segments of the liver, according to the Couinaud segmentation [47], and were automatically replicated along all multiecho images. For each ROI, the mean signal intensity across all TEs was calculated and the resulting decay curve was fitted to an exponential-plus-constant model to derive the T2* value [48].

The post-processing of native T1 and T2 images was performed using cvi42 softwareversion 5.12.1 (Circle Cardiovascular Imaging Inc., Calgary, AB, Canada). Pixel-wise T1 and T2 maps were generated using an appropriate function provided by the software and ROIs identical to those used for T2* relaxometry (same dimension and location) were manually defined in the maps. For each ROI, the T1 and T2 values were calculated as the mean of all pixel-wise T1 and T2 measurements, respectively.

A conservative approach was used in the ROI definition. All source images and maps were visually inspected for artifacts caused by susceptibility effects or motion, which could appear as focal, diffuse, or band-shaped areas of abnormal signal. Liver segments affected by significant artifacts that could compromise the accuracy of T2*, T1, or T2 measurements were excluded from analysis. No ROI was placed in liver segments that were not captured on any of the three acquired slices. Large blood vessels, bile ducts, and regions with partial volume effect, including air or perihepatic fat at the liver border, were excluded.

The caudate lobe (segment 1) could be measured in only a few cases. In the right liver, for T2*, native T1, and T2 images, segments 5 (anteroinferior) and 6 (posteroinferior) were assessable in less than 10% of the patients, while segments 7 (posterosuperior) and 8 (anterosuperior) were never visible. At least one segment in the left hepatic lobe provided good diagnostic image quality across all sequences. So, segments 2 (left lateral superior), 3 (left lateral inferior), and 4 (left medial) were selected for analysis. Global liver T2*, T1, and T2 values were obtained by averaging the respective measurements from these three left-lobe segments.

### 2.3. Laboratory Parameters

All laboratory investigations were performed at the thalassemia centers where the patients were treated. In each patient, serum hemoglobin, ferritin, and aminotransferase enzymes were assessed by commercially available kits at least 6 times a year and the mean was calculated to generate a single value per patient.

To evaluate potential disturbances of glucose metabolism, patients without a prior diagnosis of diabetes underwent an oral glucose tolerance test (OGTT) within three months of the MRI. All patients fasted overnight for a minimum of 12 h. Baseline blood samples were collected to measure glucose and insulin levels. Each patient then received a glucose solution at a dose of 1.75 g/kg, up to a maximum of 75 g, followed by measurements of glucose and insulin at 60 and 120 min.

### 2.4. Diagnostic Criteria

The lower limit of normal for the global liver T2* was 20 ms. This cut-off was evaluated considering the data of 20 healthy subjects enrolled in a previous study where several hepatic slices were acquired with a GRE sequence, with the aim of detecting all hepatic segments and measuring the T2* value in each of them [48]. The global liver T2* (mean of T2* in segments 2, 3, and 4) in this population was 26.35 ± 3.31 ms.

The normal ranges of liver T1 and T2 values for the mapping sequences employed in the present study had been previously established on a cohort of 100 healthy subjects [49]. The lower and upper limits of normal for global liver T1 values were, respectively, 442 ms and 705 ms. Since global liver T2 values were influenced by sex, sex-specific reference values were defined: 35–54 ms for males and 39–54 ms for females.

Liver cirrhosis was identified either through histological examination or by a combination of clinical and laboratory findings, along with a positive radiologic result (i.e., computerized tomography scan, MRI, ultrasonography, endoscopy) [50].

Normal glucose tolerance (NGT) was defined as a fasting plasma glucose (FPG) < 100 mg/dL and 2 h glucose < 140 mg/dL. Impaired fasting glucose (IFG) was diagnosed when FPG ranged from 100 to 126 mg/dL. Impaired glucose tolerance (IGT) was defined by 2 h plasma glucose between 140 and 200 mg/dL, with a FPG < 126 mg/dL. DM was diagnosed if FPG was ≥126 mg/dL, 2 h glucose was ≥200 mg/dL, or a random plasma glucose was ≥200 mg/dL in the presence of classic symptoms of hyperglycemia [51].

### 2.5. Statistical Analysis

All data were analyzed using SPSS version 27.0 (IBM Corp, Armonk, NY, USA) and MedCalc version 19.8 (MedCalc Software Ltd., Ostend, Belgium) statistical packages.

Continuous variables were summarized as mean ± standard deviation (SD) and categorical variables as counts and percentages. The distribution of each continuous variable was checked for normality using the Kolmogorov–Smirnov test.

To assess reproducibility, data related to 25 patients were randomly extracted from the entire dataset. These images were re-analyzed in a blinded manner by the same operator (R.B.) after an interval of at least three weeks to evaluate the intra-observer reproducibility and by a different operator (A.Co.) to evaluate the inter-observer reproducibility. Depending on data distribution, either a paired sample *t*-test or a Wilcoxon signed-rank test was used to identify significant differences between the two sets of measurements. Agreement between measurements was examined using the Bland–Altman analysis, where bias was defined as the mean difference between paired measurements and limits of agreement were calculated as the mean ± 1.96 SDs. The coefficient of variation (CoV) was calculated as the SD of the half mean square of the differences between repeated measurements divided by the overall mean. A CoV < 10% was considered indicative of good reproducibility. The intraclass correlation coefficient (ICC) was obtained from a two-way random effects model with measures of absolute agreement. ICC values ≥ 0.75 were interpreted as excellent reliability, values between 0.40 and 0.75 as good, and values < 0.40 as poor.

Group comparisons were conducted using either the independent-samples *t*-test or the Mann–Whitney test for continuous variables, while the χ^2^ testing was employed for categorical variables.

Correlation coefficients (R) and corresponding *p*-values were calculated to assess the strength and significance of associations between variables. For normally distributed data, Pearson correlation coefficients were used, while Spearman rank correlation coefficients were applied for non-normally distributed data.

The cocor-package of R (http://comparingcorrelations.org/) was employed to compare the strength of two overlapping correlations, in which one variable was shared between both correlation pairs [52].

The receiver operating characteristic (ROC) analysis was used to assess the ability of the hepatic relaxation times to discriminate the presence of a specific condition and the results were presented as areas under the curve (AUCs) with 95% confidence intervals (CIs). An AUC value of 0.5 indicated no better-than-chance discrimination, whereas an AUC of 1.0 reflected perfect predictive performance. The optimal cut-off value was determined using the Youden index. Differences between AUCs were evaluated using DeLong’s test.

In all tests, a two-sided *p*-value of 0.05 was considered the threshold for significance.

## 3. Results

### 3.1. Patients’ Characteristics

We considered 81 TDT patients (52 females, 38.13 ± 10.79 years). All patients were regularly transfused since early childhood to maintain a pre-transfusion hemoglobin concentration above 9–10 g/dL and were chelated.

Demographic, clinical, laboratory, and instrumental characteristics of the patients are summarized in Table 1.

Mean global liver T2* value was 13.34 ± 9.55 ms (range: 1.52–36.11 ms). A reduced global liver T2* value was found in 59 (72.8%) patients.

Mean global liver T1 value was 441.29 ± 75.01 ms (range: 335.47–666.43 ms). A reduced global liver T1 value was found in 48 (59.3%) patients, while no patient had an increased global liver T1 value.

Mean global liver T2 value was 37.84 ± 7.98 ms (range: 16.91–64.75 ms). Forty-three (53.1%) patients showed a reduced global liver T2 value, while only one (1.2%) patient presented with an increased global liver T2 value.

### 3.2. Reproducibility of Hepatic T2*, T1, and T2 Values

Table 2 and Table 3 show the results of the intra- and inter-observer reproducibility for segmental and global T2*, T1 and T2 values. For all relaxation times, the paired test showed no significant differences. The Bland–Altman analysis showed no systematic differences between assessments. The CoV was always <10% and the ICC was always excellent.

### 3.3. Association Among the Three Liver Relaxation Times

**Figure 1:**
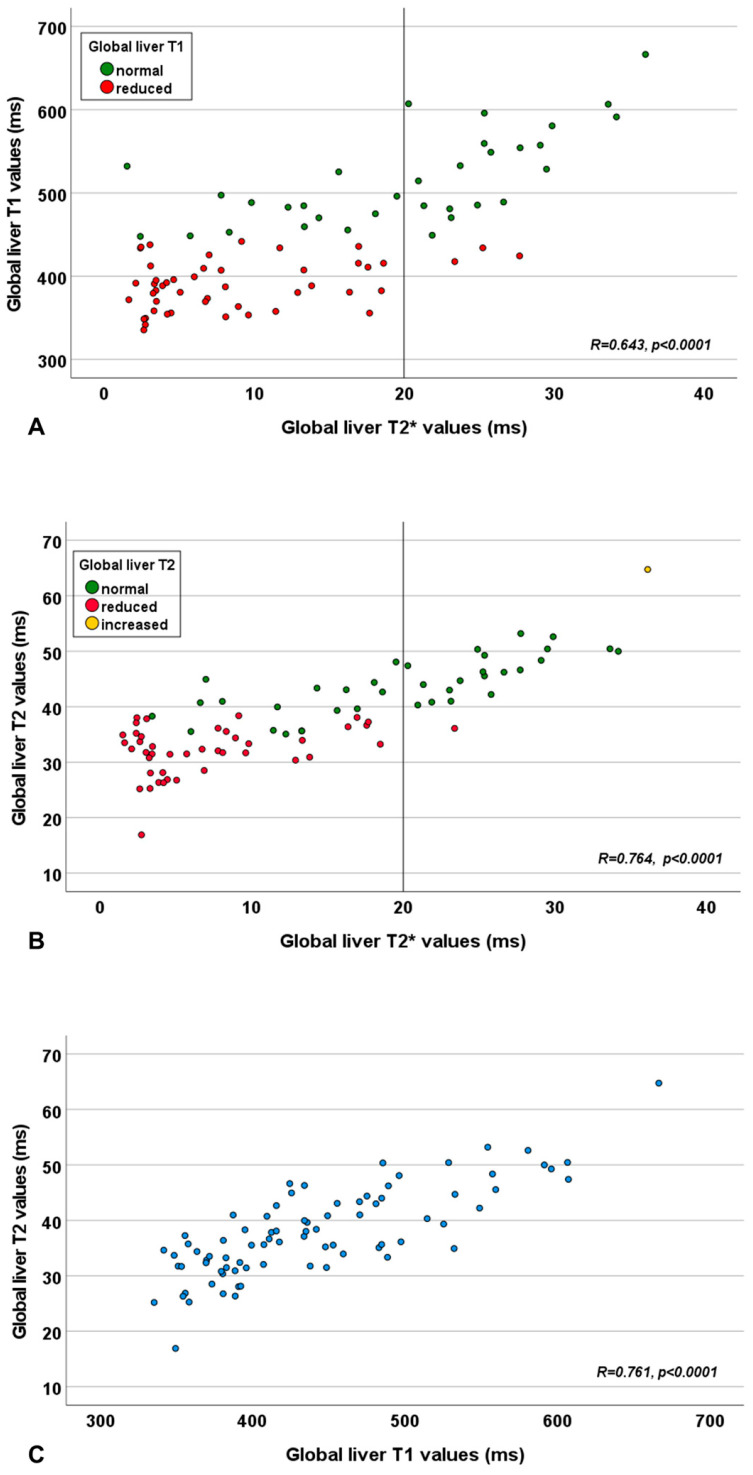
Scatter plot showing the relationship between global liver T2* and T1 values (**A**), global liver T2* and T2 values (**B**), and global liver T1 and T2 values (**C**). The vertical line in panels (**A**,**B**) represents the cut-off for the global liver T2* value.

A significant correlation was detected between global liver T2* and T1 values, T2* and T2 values, and T1 and T2 values (Table 1 and Figure 1). The correlation between global liver T2* and global liver T2 values was significantly stronger than that between T2* and T1 values (*p* = 0.018).

Among the 59 patients with a reduced global liver T2* value, 45 (76.3%) had also a reduced T1 value, while 42 (71.2%) had a reduced global liver T2 value. Among the 22 patients with a normal global liver T2* value, 3 (13.6%) patients exhibited a reduction in any of the two other relaxation times. Specifically, two patients had a reduced T1 value and one patient showed a reduction in both T1 and T2 values.

### 3.4. Demographic and Clinical Correlates of Liver Relaxation Times

Table 1 shows the correlation of the three liver relaxation times with demographic, biochemical, and clinical parameters.

No association was detected between gender or splenectomy and liver relaxation times. Global liver T2* and T2 values were independent of age, while global liver T1 values significantly increased with age.

Only one patient had a chronic HCV infection (positive HCV antibodies and HCV ribonucleic acid), while 49 patients had a past HCV infection, eradicated spontaneously or after treatment with antiviral therapy. No difference was found in liver relaxation times between negative patients and patients with past or active HCV infection.

All three liver relaxation times exhibited a significant inverse correlation with serum ferritin levels.

Global liver T2 and T2* values were inversely correlated with mean alanine aminotransferase levels, while no relaxation time correlated with mean aspartate aminotransferase or gamma-glutamyl transferase levels.

### 3.5. Liver Relaxation Times and Complications

**Figure 2:**
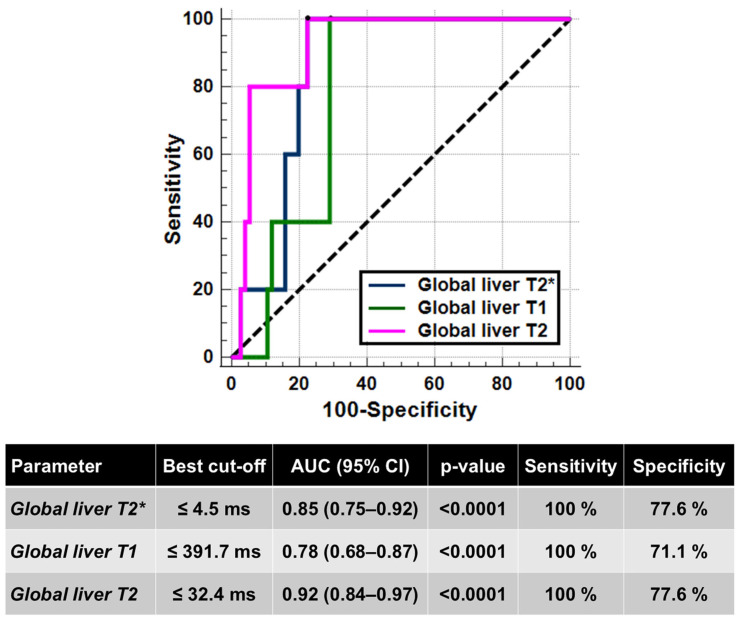
ROC curve analysis of liver relaxation times to identify the presence of hepatic cirrhosis.

Five (6.2%) patients had hepatic cirrhosis. Compared to patients without cirrhosis, patients with cirrhosis had significantly lower global liver T2* values (3.42 ± 0.87 ms vs. 13.99 ± 9.50 ms; *p* = 0.010), T1 values (337.08 ± 18.26 ms vs. 445.52 ± 75.44 ms; *p* = 0.036), and T2 values (27.79 ± 2.77 ms vs. 38.49 ± 7.77 ms; *p* = 0.002), while age was not significantly different (44.62 ± 7.36 years vs. 37.70 ± 7.36 years; *p* = 0.158). No patient with cirrhosis had a normal relaxation time, suggesting a negative predictive value of 100% of all hepatic relaxation times for cirrhosis. One patient with cirrhosis had never contracted the HCV infection. Figure 2 shows the ROC curves and the best cut-offs of hepatic relaxation times for predicting the presence of hepatic cirrhosis. All three relaxation times were able to discriminate between the absence and the presence of cirrhosis (T2*: AUC = 0.85; T1: AUC = 0.78; T2: AUC = 0.92). The T2* showed a discriminatory ability comparable to that of T1 and T2 values (*p* = 0.417 and *p* = 0.344, respectively), while a significant difference was found between the AUCs of T1 and T2 values (*p* = 0.011).

**Figure 3:**
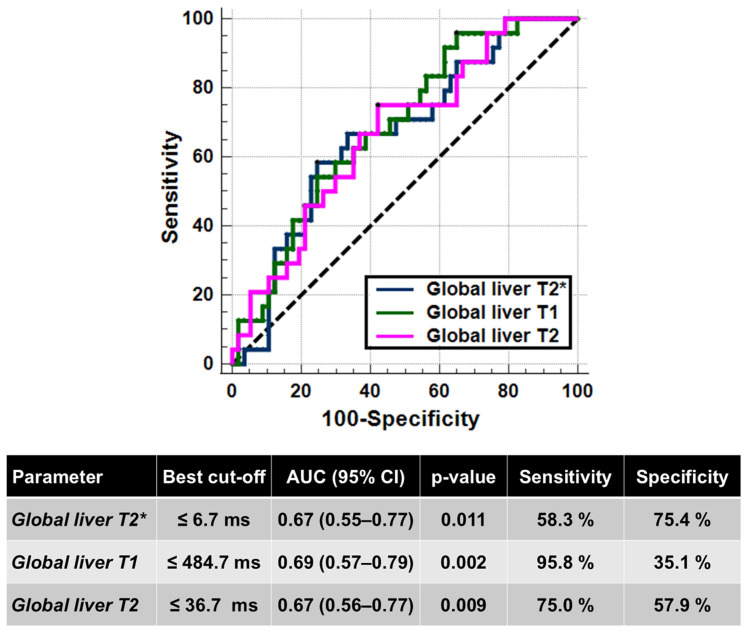
ROC curve analysis of liver relaxation times to identify the presence of alterations in the glucose metabolism.

Twenty-four (29.6%) patients had an altered glucose metabolism: 2 IFG, 8 IGT, and 14 DM. Compared to patients with normal glucose metabolism, patients with an altered glucose metabolism showed significantly lower global liver T2* values (9.47 ± 7.77 ms vs. 14.97 ± 9.58 ms; *p* = 0.019), T1 values (406.92 ± 50.17 ms vs. 455.77 ± 79.28 ms; *p* = 0.008), and T2 values (34.32 ± 11.29 ms vs. 39.32 ± 7.99 ms; *p* = 0.017). Figure 3 shows the ROC curves and the best cut-offs of hepatic relaxation times for predicting the presence of an altered glucose metabolism. All three relaxation times were able to discriminate between the absence and the presence of alterations of glucose metabolism (T2*: AUC = 0.67; T1: AUC = 0.69; T2: AUC = 0.67). The Delong’s test did not show a significant difference among the AUCs (T2* vs. T1: *p* = 0.701; T2* vs. T2: *p* = 0.948; T2 vs. T1: *p* = 0.715).

## 4. Discussion

Quantitative MRI assessment is gradually proving to be a complementary tool, if not even a future replacement, for qualitative MRI sequences in the study of pathological alterations of the liver, given its ability to provide accurate and standardized data [53]. In TDT patients, quantitative MRI has already established itself as the gold standard in evaluating iron deposits in the liver and other body organs [31,32,33,34,35,36].

All three relaxation times demonstrated minimal intra-observer and inter-observer variability, ensuring that measurements remain consistent and reliable across different operators and scanning sessions.

Nearly three-quarters of our TDT cohort (72.8%) showed reduced hepatic T2* values (<20 ms). This is consistent with previous studies, which, despite using varying cut-off thresholds, have consistently reported a high prevalence of reduced T2* in TDT populations [9,54,55]. We also found that a substantial proportion of patients had reduced liver T1 (59.3%) and T2 (53.1%) values compared with healthy controls examined on the same MRI platform [49]. The fact that more than one-quarter of patients with reduced global liver T2* had normal T1 and T2 values suggests that these parameters may be less accurate markers of hepatic iron overload. This pattern could reflect the influence of concurrent processes—such as inflammation or edema—that counteract the relaxation-shortening effect of iron deposition, thereby masking the true extent of iron burden on T1 and T2 mapping. In non-thalassemic populations, liver fibrosis [56] and steatosis [57] have been linked to elevated T1 values. Native T1 can increase due to a higher extracellular water fraction, as in inflammatory edema [58], or due to extracellular matrix expansion, characteristic of fibrosis and cirrhosis [59]. For T2 relaxation, evidence points to a complex interplay among expanded extracellular fibrous matrix, inflammatory or congestive edema, and inflammatory cell infiltration, all contributing to increased values [60]. However, its role in liver tissue characterization remains debated [61]. Animal studies suggest T2 correlates more strongly with inflammation than fibrosis [62], and that T1 may outperform T2 in reflecting collagen deposition in the liver [63].

Anyway, the detection of reduced T1 values despite normal T2* measurements in three patients suggests that T1 mapping can serve as a complementary tool for assessing early or subtle iron overload that may not yet be captured by T2*. This can be due to the acquisition technique commonly used in clinical practice, which loses accuracy when measuring T2* values outside the typical clinical range. Specifically, the signal decay is only partially sampled because of the relatively low maximum TE, which can limit T2*’s sensitivity to mild iron accumulation [64].

Liver T2* values in our cohort were inversely correlated with serum ferritin levels, in line with current evidence [48,65,66,67]. T1 and T2 values demonstrated a similar relationship with ferritin, consistent with earlier findings using T1 and T2 signal intensity ratio (SIR) techniques in TDT patients [68]. Both T2 and T2* values were also inversely associated with alanine aminotransferase levels, although previous literature supports this association only for T2* [66,67]. In the general population, higher serum ferritin has been linked to greater severity of steatosis, inflammation, and fibrosis in MASLD [69], and to increased mortality in both MASLD [70] and decompensated cirrhosis [71]. In thalassemia, however, this relationship appears more complex: ferritin correlates with triglyceride levels [72] and Fibroscan scores [73], while hepatic steatosis can attenuate the association between ferritin and liver iron concentration [74,75]. These findings suggest a multifaceted interplay between iron overload, liver injury, and lipid metabolism.

A small fraction of the study population with a confirmed diagnosis of liver cirrhosis (5 patients, 6.2%) had reduced T2*, T1, and T2 values. Iron overload has been shown to be related to fibrosis and subsequent cirrhosis [27]. Importantly, all three relaxation parameters demonstrated significant discriminatory ability to differentiate cirrhotic from non-cirrhotic patients, albeit with only moderate specificity. This suboptimal specificity implies a potential for false-positive results, thereby limiting the utility of these measures as standalone diagnostic tools in clinical settings. However, the significant difference observed between T1 and T2 AUCs (*p* = 0.011) suggests that T2 may provide superior discriminatory capability over T1 in this context. The T1 cut-off identified in our study for predicting cirrhosis (≤391.7 ms) is substantially lower than thresholds reported in non-thalassaemic populations. For instance, a study involving 200 patients without iron overload established a cirrhosis cut-off of >624 ms, albeit with modest sensitivity (60%), and reported significantly elevated T1 values (653 ± 62 ms) in biopsy-confirmed cirrhosis (METAVIR stage F4) [76]. Similarly, two other studies on 109 and 68 non-iron-overloaded patients, combining T1 mapping with MR Elastography, identified T1 thresholds for cirrhosis of >592.5 ms and >940 ms, respectively, with comparable specificities (76% and 75%) [77,78]. These findings suggest that iron overload exerts a predominant effect on T1 relaxation times in TDT patients, likely overshadowing influences from edema, fibrosis, or steatosis. Similarly, the T2 cut-off of ≤32.4 ms proposed in our study to predict cirrhosis differs substantially from the >52 ms threshold reported in a recent study of 75 non-thalassaemic patients with chronic liver disease and 25 healthy controls [79]. Another investigation involving 123 patients without iron overload demonstrated a significant correlation between elevated T2 values (72.4 ± 1.4 ms) and severe fibrosis and cirrhosis (Ishak grades 5 and 6) [42]. Reduced T2 relaxation time among TDT patients with cirrhosis may suggest that iron overload could be an additional factor to consider together with inflammatory edema, and the degree of influence of each factor should be better outlined. Overall, the small number of cirrhotic patients in the reference population limits our conclusions, and further evidence should be collected on a larger sample.

Altered glucose metabolism is a hallmark complication in TDT, driven by a complex interplay of insulin deficiency and resistance. Insulin deficiency primarily results from iron deposition within pancreatic beta cells, while insulin resistance is linked to hepatic iron overload, which impairs insulin’s capacity to suppress hepatic glucose production, as well as to iron accumulation in skeletal muscle that reduces glucose uptake [80,81,82]. In our cohort, patients presenting with altered glucose metabolism exhibited significantly reduced T2*, T1, and T2 liver relaxation times, suggesting greater hepatic iron burden and tissue alterations. All relaxation times demonstrated similar diagnostic accuracy in differentiating patients with altered glucose metabolism from those without; however, the identified cut-off values failed to achieve both high sensitivity and specificity simultaneously. This suggests that liver MRI metrics alone may not be sufficient to fully characterize the complex disturbances in glucose metabolism observed in this population. Combining hepatic and pancreatic iron quantification may provide a more comprehensive understanding of the pancreas-liver metabolic axis and may enhance stratification of TDT patients [83].

### 4.1. Study Limitations

T1 and T2 relaxation values are known to vary depending on the MRI scanner used, highlighting the need to establish scanner-specific normal reference ranges before applying these metrics to assess pathological conditions. This study has a retrospective design and the mapping sequences analyzed were not specifically optimized for liver imaging. Consequently, since the image planes were not axial to the liver, some measurement inaccuracies and potential misattribution of values to specific hepatic segments cannot be excluded. Additionally, for certain patients, the multiparametric evaluation was restricted to a single hepatic segment due to suboptimal image quality in others.

### 4.2. Future Directions

Further refinement and validation of T1 mapping techniques are needed to better stratify fibrotic risk and accurately identify cirrhosis within the TDT population. Advanced MRI approaches, such as Dixon water-only fat-corrected MOLLI sequences, which reduce the confounding effects of hepatic steatosis [84], and fat-suppressed inversion recovery spin-echo echo-planar imaging protocols, which minimize sensitivity to both iron and fat [85], hold promise in this regard. Moreover, iron-corrected T1 mapping algorithms designed to compensate for iron interference may enhance diagnostic precision [86,87].

## 5. Conclusions

This study supports the important role of T2* mapping in liver iron quantification and suggests potential benefits of including T1 and T2 quantitative tissue characterization in standard MRI protocols. Such integration may provide valuable, non-invasive insights into liver disease and associated metabolic disturbances, ultimately supporting improved monitoring and management of complications in TDT. Future research should further explore these parameters to clarify their utility in the early detection and risk stratification of hepatic and metabolic complications in this patient population.

## Figures and Tables

**Table 1:** Demographic, clinical, biochemical, and MRI characteristics of TDT patients and correlations with global liver T2*, T1, and T2 values.

		Global Liver T2* Values	Global Liver T1 Values	Global Liver T2 Values
Categorical Variable				
	Frequency, *N* (%)	Difference in relaxation times between two groups (absent vs. present)		
Female sex	52 (64.2)	14.19 ± 9.52 ms vs. 12.86 ± 9.63 ms (*p* = 0.497)	458.65 ± 80.21 ms vs. 431.61 ± 70.90 ms (*p* = 0.129)	39.11 ± 7.46 ms vs. 37.13 ± 8.24 ms (*p* = 0.279)
Splenectomy	32 (39.5)	13.24 ± 9.18 ms vs. 13.49 ± 10.25 ms (*p* = 0.988)	434.78 ± 68.07 ms vs. 451.27 ± 84.72 ms (*p* = 0.417)	37.22 ± 7.24 ms vs. 38.79 ± 9.03 ms (*p* = 0.364)
Past/active HCV infection	50 (61.7)	12.46 ± 9.59 ms vs. 12.88 ± 9.59 ms (*p* = 0.553)	430.72 ± 75.59 ms vs. 447.97 ± 74.62 ms (*p* = 0.252)	37.13 ± 8.96 ms vs. 38.28 ±7.37 ms (*p* = 0.627)
Continuous variables				
	Mean value	Correlation (R, *p*-value) with relaxation times		
Age	38.13 ± 10.79 years	R = 0.161, *p* = 0.150	R = 0.264, *p* = 0.017	R = 0.178 *p* = 0.112
Age at start of regular transfusions	10.74 ± 8.67 months	R = 0.149, *p* = 0.401	R = 0.262, *p* = 0.134	R = 0.098, *p* = 0.582
Pre-transfusion hemoglobin	9.71 ± 0.42 g/dL	R = 0128, *p* = 0.319	R = −0.027, *p* = 0.832	R = −0.089, *p* = 0.486
Mean serum ferritin	845.23 ± 746.01 ng/mL	R = −0.545, *p* < 0.0001	R = −0.423, *p* = 0.001	R = −0.662, *p* < 0.0001
Mean alanine aminotransferase	21.87 ± 10.22 U/L	R = −0.309, *p* = 0.017	R = −0.157, *p* = 0.236	R = −0.320, *p* = 0.013
Mean aspartate aminotransferase	21.68 ± 7.27 U/L	R = −0.186, *p* = 0.163	R = −0.040, *p* = 0.767	R = −0.179, *p* = 0.179
Mean gamma-glutamyl transferase	17.80 ± 12.01 U/L	R = −0.220, *p* = 0.161	R = 0.192, *p* = 0.224	R = 0.022, *p* = 0.892
Global liver T2* values			R = 0.643 *p* < 0.0001	R = 0.764, *p* < 0.0001
Global liver T1 values		R = 0.643, *p* < 0.0001		R = 0.761, *p* < 0.0001
Global liver T2 values		R = 0.764, *p* < 0.0001	R = 0.761, *p* < 0.0001	

*N* = number; HCV = hepatitis C virus.

**Table 2:** Intra-observer reproducibility of hepatic T2*, T1, and T2 measurements.

Region	Intra-Operator				
	Paired Test		Bland–Altman Limits (ms)	CoV (%)	ICC
	Mean Difference (ms)	*p*-Value			
T2* values					
Segment 2	0.14 ± 0.63	0.223	−1.11 to 1.38	3.76	0.999
Segment 3	0.17 ± 0.67	0.587	−1.13 to 1.47	4.49	0.998
Segment 4	−0.45 ± 1.07	0.241	−2.55 to 1.65	5.27	0.994
Global	0.06 ± 0.61	0.543	−1.13 to 1.25	3.53	0.999
T1 values					
Segment 2	6.19 ± 28.66	0.367	−49.98 to 62.37	4.59	0.974
Segment 3	2.31 ± 33.36	0.231	−63.09 to 67.70	5.29	0.967
Segment 4	0.02 ± 21.33	0.721	−41.79 to 41.83	3.25	0.992
Global	4.33 ± 18.31	0.158	−31.55 to 40.22	2.98	0.989
T2 values					
Segment 2	−0.62 ± 2.46	0.142	−5.45 to 4.21	4.72	0.983
Segment 3	0.18 ± 2.55	0.616	−4.83 to 5.18	4.87	0.983
Segment 4	0.78 ± 2.29	0.314	−3.71 to 5.26	4.23	0.979
Global	−0.11 ± 1.18	0.983	−2.41 to 2.20	2.23	0.996

CoV = coefficient of variability; ICC = intraclass correlation coefficient.

**Table 3:** Inter-observer reproducibility of hepatic T2*, T1, and T2 measurements.

Region	Inter-Operator				
	Paired Test		Bland–Altman Limits (ms)	CoV (%)	ICC
	Mean Difference (ms)	*p*-Value			
T2* values					
Segment 2	0.02 ± 0.86	0.808	−1.68 to 1.69	4.95	0.997
Segment 3	0.19 ± 0.83	0.407	−1.44 to 1.82	4.59	0.996
Segment 4	−0.29 ± 1.10	0.721	−2.45 to 1.87	5.17	0.994
Global	0.03 ± 0.69	0.661	−1.33 to 1.39	3.99	0.998
T1 values					
Segment 2	−5.41 ± 29.40	0.443	−63.04 to 52.22	4.62	0.974
Segment 3	5.42 ± 32.21	0.216	−57.72 to 68.56	5.18	0.967
Segment 4	−8.83 ± 19.99	0.386	−48.01 to 30.35	3.33	0.991
Global	−0.95 ± 20.21	0.882	−40.57 to 38.66	3.18	0.988
T2 values					
Segment 2	−0.34 ± 2.89	0.647	−6.01 to 5.33	5.42	0.978
Segment 3	0.98 ± 2.42	0.184	−3.76 to 5.72	5.05	0.981
Segment 4	0.82 ± 2.60	0.477	−4.28 to 5.92	4.77	0.972
Global	0.32 ± 1.75	0.632	−3.10 to 3.74	3.37	0.991

CoV = coefficient of variability; ICC = intraclass correlation coefficient.

## Data Availability

The data presented in this study are available on request from the corresponding author. The data are not publicly available due to privacy.
